# Identification and characterization of learning weakness from drawing analysis at the pre-literacy stage

**DOI:** 10.1038/s41598-022-26038-9

**Published:** 2022-12-14

**Authors:** Linda Greta Dui, Eugenio Lomurno, Francesca Lunardini, Cristiano Termine, Alessandro Campi, Matteo Matteucci, Simona Ferrante

**Affiliations:** 1 Department of Electronics, Information and Bioengineering, Politecnico di Milano, Via Ponzio 34/5, 20133 Milan, Italy; 2 Child Neuropsychiatry Unit, Fondazione IRCCS Istituto Neurologico Carlo Besta, Milan, Italy; 3 Child Neuropsychiatry Unit, Department of Medicine and Surgery, University of Insubria, Varese, Italy

**Keywords:** Biomedical engineering, Disease prevention

## Abstract

Handwriting learning delays should be addressed early to prevent their exacerbation and long-lasting consequences on whole children’s lives. Ideally, proper training should start even before learning how to write. This work presents a novel method to disclose potential handwriting problems, from a pre-literacy stage, based on drawings instead of words production analysis. Two hundred forty-one kindergartners drew on a tablet, and we computed features known to be distinctive of poor handwriting from symbols drawings. We verified that abnormal features patterns reflected abnormal drawings, and found correspondence in experts’ evaluation of the potential risk of developing a learning delay in the graphical sphere. A machine learning model was able to discriminate with 0.75 sensitivity and 0.76 specificity children at risk. Finally, we explained why children were considered at risk by the algorithms to inform teachers on the specific weaknesses that need training. Thanks to this system, early intervention to train specific learning delays will be finally possible.

## Introduction

The first years of primary school represent a delicate period in which children build the basis of their knowledge and their whole life. Difficulties in the learning process must be promptly addressed, as they can affect whole children’s lives, resulting in early school abandon, behavioral problems ^1^, or criminal attitudes ^2^. Even in the digital era, handwriting is a fundamental skill for school-aged children ^3^, but children who struggle with mastering it are comprised between 5 and 27% of the school-aged population ^4,5^. This can be traced back to handwriting complexity, that involves different sub-domains, such as dexterity, eye-hand coordination, programming capabilities, and automation ^6^. As handwriting is still a fundamental mean for self-expression, difficulties in learning this skill are particularly frustrating. It is then recognized that an early intervention helps to lower the number of children in difficulty ^7^, and responsiveness to treatments can also be considered a distinctive trait between a simple handwriting delay and a more severe learning disability.

The specific learning disability associated with handwriting impairments is called dysgraphia, and it is characterized by handwriting skills below the expectancy nonetheless age, intelligence, and absence of sensori-motor problems ^6^. Even though an early diagnosis is fundamental, it is often delayed for different reasons. First, guidelines require that handwriting should be tested when mature, i.e., between second and third grade ^5^. Second, in-person visits with child neuropsychiatrists may not be easily accessible due to factors such as the long waiting lists and the cost. A situation that has been exacerbated by the COVID-19 pandemic ^8^. In the meantime, the responsibility of helping children is shifted to teachers, who may observe an abnormal learning path and suggest reinforcement exercises. Notwithstanding the important role of teachers, their observation is subjective and experience-dependant, as schools lack specialized professionals. Therefore, some children with a learning delay might be left apart until the exacerbation of symptoms brings to severe consequences. Additionally, school closure during the COVID-19 lockdown made a direct observation impossible, stressing the limitation of such approach. On the other hand, implementing preventive training programs for entire classrooms is not feasible, as they are time consuming and costly due to teachers’ training.

To overcome these limits, novel screening approaches must include the possibility to objectively study the learning trajectory of children from an early stage, even in a remote setting. The digitalization of dysgraphia diagnosis has been a trending topic during the last years ^9–12^. The standard approach includes a digital tool—such as, a digitizer or a tablet equipped with a digital pen, or a smart ink pen to write directly on paper ^13^—capable of collecting information about pen pressure and tilt. Recruited subjects are asked to write some grapheme, word or sentence, and both the written trace and various extracted features are correlated to handwriting proficiency. The latter is usually estimated through standard handwriting tests, such as the BVSCO-2 or the BHK ^14,15^. Both classical statistics and novel machine learning models have been employed to characterize the differences between typically developing and dysgraphic handwriting. Handwriting features that were found to be informative in the discrimination between proficiency levels can be grouped into macro-areas, that are: fluidity-related features, such as the signal-to-noise velocity peak difference ^16^, or the time spent with the pen lifted from the sheet ^17^; pen angle-related features, such as the variability of the tilt ^18^; pressure-related features, such as the pressure frequency content or variability  ^9^; time-related features, such as the time to write in an accurate way (speed-accuracy trade off) ^19^, or the time adjustments related to writing size (isochrony and homothety) ^20^. Even though a digital evaluation can ease the diagnosis process by improving objectiveness and allowing a remote assessment, this approach has the same weakness described for standard dysgraphia tests, i.e., it relies on handwriting knowledge, and does not allow to anticipate the screening.

An intuition to allow moving dysgraphia screening to a pre-literacy stage is to leverage the same parameters that have been studied for handwriting, but applying them to symbol drawing. This is the rationale behind the *Play-Draw-Write* tablet app ^21^. *Play-Draw-Write* comprises a *Copy Game* and a *Tunnel Game*. The game design is based on handwriting laws known to be altered in dysgraphic handwriting, that are isochrony, homothety and speed-accuracy trade off. Isochrony and homothety, together, assure that absolute and relative (letter-wise) writing time is kept constant, even when writing bigger or smaller. Speed-accuracy trade off predicts a linear relationship between the difficulty of a task and the time needed to perform it. Instead of investigating handwriting alteration, the game focuses on symbol drawing: to study isochrony, the request is to draw bigger or smaller symbols; to study homothety, the request is to draw a sequence of symbols at different sizes; to study the speed-accuracy trade off, the request is to steer letter- and symbol-shaped paths. Playing on a tablet equipped with a smart pen enables the extraction of pen position, pressure and angles in time, which provide information about gesture production. Such objective parameters can be processed to match the features that proved to be powerful in discriminating between typically developing and dysgraphic handwriting. Leveraging symbols instead of words makes the tool independent from language. Moreover, the gamification of such tests assures better adherence in young children, and the wide availability of tablets makes the app more suited for a pre-clinical screening compared to digitizers ^21^. The challenge of this approach is to leverage game performance to infer the potential difficulty that a child is encountering at the very beginning of its learning path, nonetheless the intrinsic uncertainty of defining such difficulty.

The aim of this work is to leverage drawings to automatically suggest directions for potential handwriting weaknesses remediation when handwriting is not learned yet. To achieve this goal, we can identify three sub-aims: (1) to assess the validity of the proposed tool in providing an objective evaluation of children’s graphical abilities; (2) to detect which children mostly need to undergo a training program; (3) to locate the areas where children mostly need to be empowered.

## Results

### Participants

A total of 241 children attending the last year of Kindergarten completed the evaluation by playing the four *Play-Draw-Write* games: *Copy Game-Square* (CGSq), *Copy Game-Sequence* (CGSe), *Tunnel Game-Square* (TGS), and *Tunnel Game-Word* (TGW). See Supplementary Figure S1 for games examples. Median age was 5.6, quartiles [5.3, 5.7]. 232 preferentially drew with the right hand, 9 with the left. 124 were male, 117 female. 165 were Italian mother-tongue, 76 were not.

### Risk evaluation by teachers

**Figure 1:**
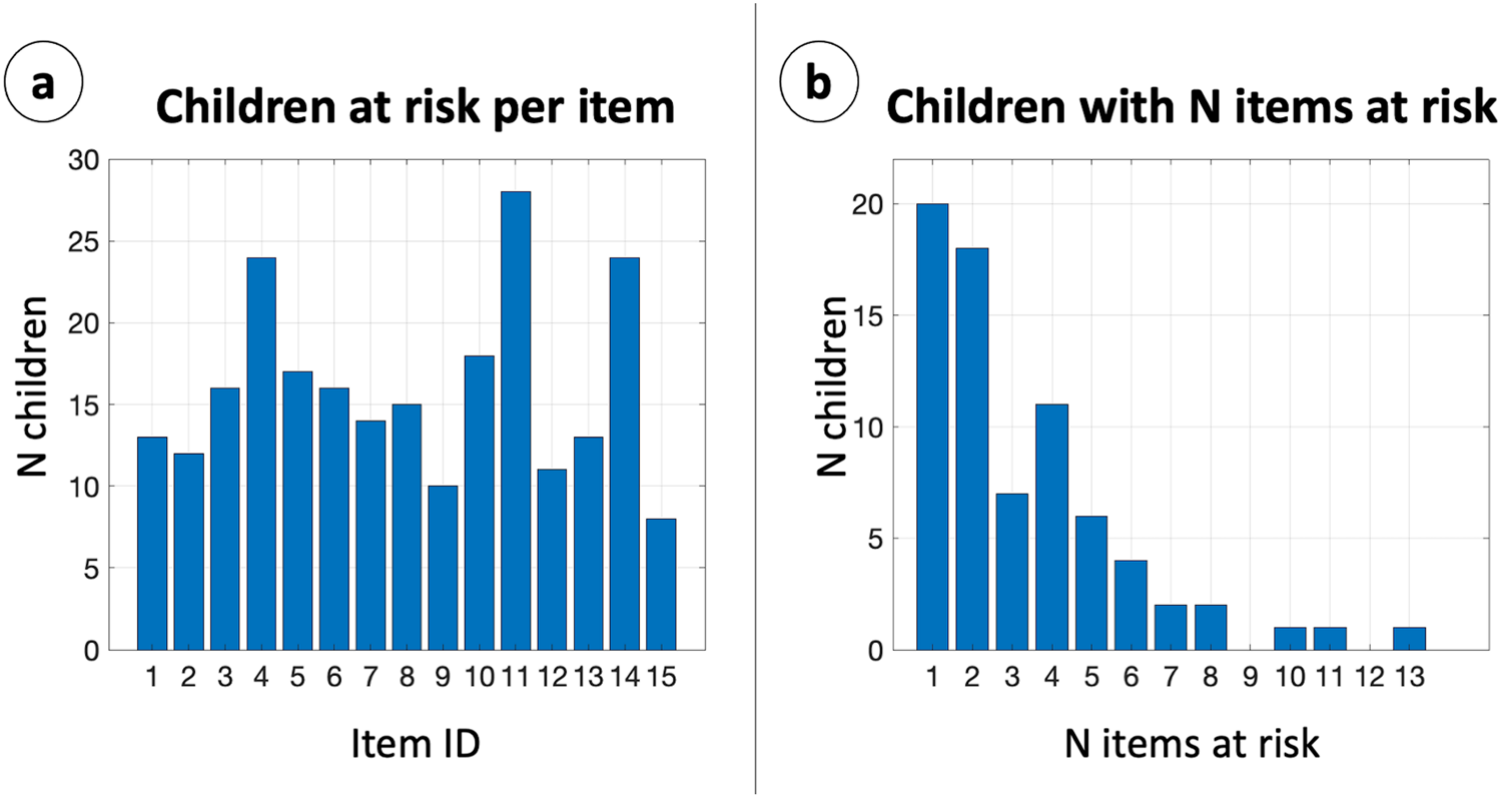
Children at risk. (**a**) X: item of the checklist; Y: number of children at risk for a given item. (**b**) X: number of items selected for a child; Y: number of children with a given number of items considered at risk.

The risk of a grapho-motor delay was assessed by trained teachers, according to the 15-items checklist reported in Supplementary Methods S2. Figure 1 reports the number of children deemed at risk for a specific item of the checklist (Panel a), and the number of children with a given number of items at risk (Panel b). 73 children out of 241 (30.29%) were considered at risk in at least one item. We considered these children as the Risk (R) group, whilst the others are the No-Risk (NR) group. Between R and NR groups, we did not find differences in age ($p=0.957$), nor in the proportion of right/left-handers ($p=0.220$); however, the relative number of males in the R group was significantly higher (NR: 46.4%; R: 64.4%; $p=0.010$), as well as the relative number of non-Italian mother-tongue (NR: 23.8%; R: 42.5%; $p=0.004$).

9.52% of the Spearman’s correlation coefficients between items responses were comprised between 0.3 and 0.7, 8.57% were lower than 0.3, 81.91% were not significant. All significant correlations were positive. In particular, Items 13 (writing oneself’s name) and 15 (distinguishing letters from different graphical signs) had a correlation higher than 0.5.

### Graphical abilities analysis

To assess the validity of the *Play-Draw-Write* solution in evaluating children’s graphical abilities, we leveraged the features extracted from the games in an unsupervised way, independently from teachers’ evaluation. This approach is inspired by classical dysgraphia tests ^14,15^, where normative ranges are built based on the average performance of the age-matched population. Then, children who deviate from their peers (e.g., two standard deviations lower than the mean writing speed ^14^) are considered affected by dysgraphia. We hypothesise that the same would apply to game performances and to the features extracted from the *Play-Draw-Write* app. Hence, we detected “outlier children” by computing an “outlier score” (O-score), as defined in the "Methods" section. The O-score is expected to be low if a child’s performance is aligned with the majority of peers, high if it differs, thus revealing a potential risk.

**Figure 2:**
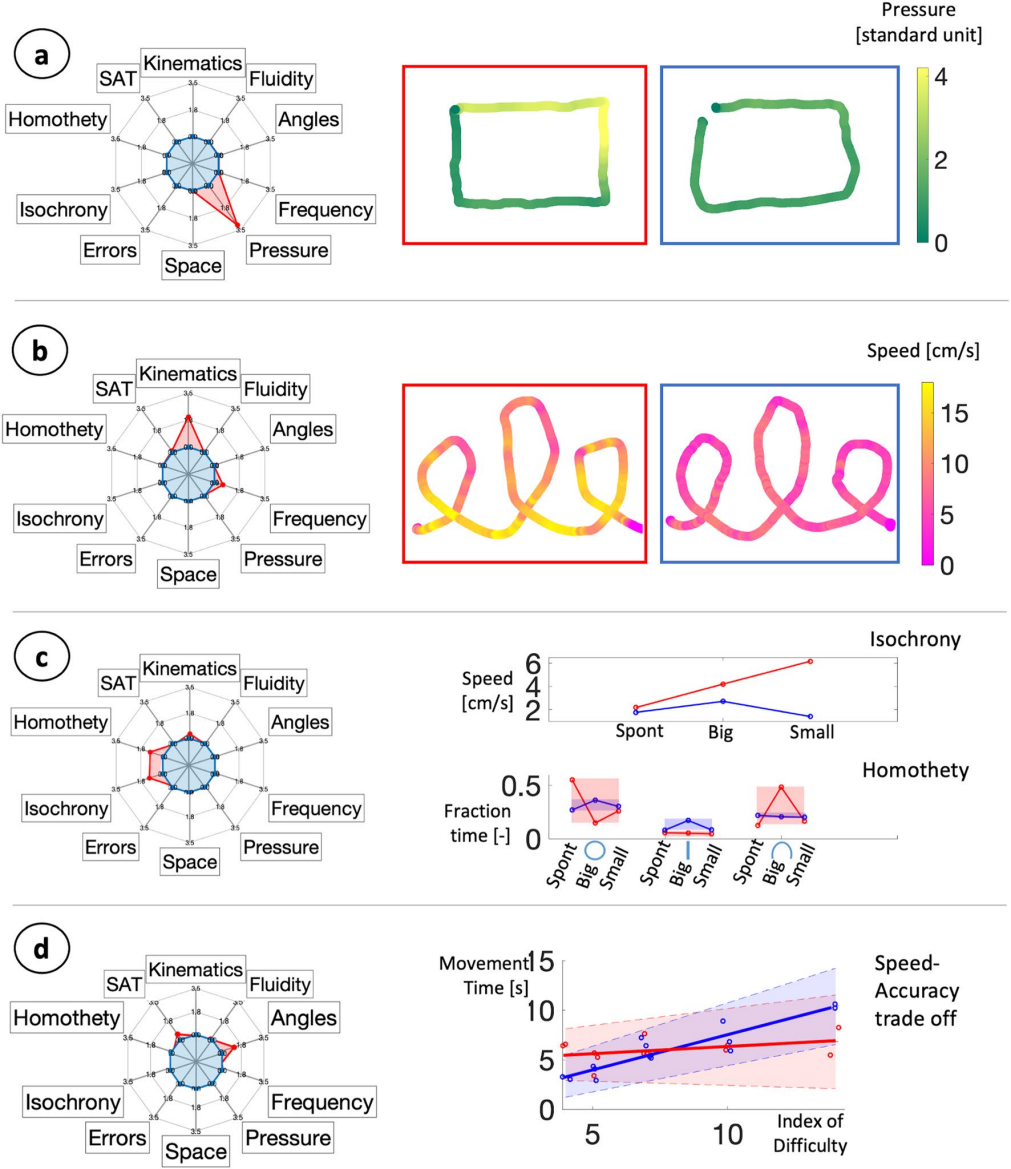
O-score correspondence with children executions. (**a–d**) report in red a sample subject with a high (i.e., bad) O-score in a specific feature category; in blue a sample subject with a low O-score. Axes of the radar plot show the O-score divided by feature category, computed only on the game shown in the right part of the Panels. (**a**) Pressure from TGS, colours correspond to pressure. (**b**) Kinematics from the TGW, colours correspond to execution speed. (**c**) Isochrony and Homothety from CGSe, speed and fraction time of spontaneous, big and small executions are shown. (**d**) Speed-Accuracy trade off from the TGS.

Figure 2 represents some examples of the O-score, together with the related execution. Radar plots were used to display the O-score obtained in each game, stratified by feature category, as defined in the "Methods" section. Panel a, in the red box, shows a subject who applied an oddly high pressure, compared to the subject in the blue box, and this is reflected into a high (i.e., bad) O-score in the Pressure category for the TGS. Panel b, in the red box, shows a subject that drew with frequent changes in speed. This behaviour is reflected into a high O-score in the Kinematics category for the TGW. The subject reported in the blue box, instead, performed the task with a more homogeneous speed (O-score equal to zero in all feature categories). Panel c compares both isochrony and homothety in two subjects who scored high (red) and low (blue) in the corresponding O-score. From the speed plotted against symbols size, we can notice that the expected modulation was not present in the subject who scored high in the Isochrony feature category. In fact, when writing smaller, a lower speed would have been expected to keep execution time approximately constant, as in the blue subject. On the contrary, the red subject drew faster when asked to draw small, thus reducing the execution time. A similar trend can be observed in the rest of the population. Unexpected speed modulation (big speed < spontaneous speed; big speed < small speed; small speed > spontaneous speed) were more frequent in children who achieved an O-score greater than zero in the Isocrony feature category (CGSq: 53.1%; CGSe: 40.2%; Chi-squared test *p*-value < 0.001 for both games) than in those who had an O-score equal to zero (CGSq: 16.1%; CGSe: 15.4%).

As for homothety, the fraction time in Fig. 2c (i.e., the time to draw each symbol, divided by the total time to draw the sequence) was more variable in the subject who obtained an O-score greater than zero in the Homothety feature category. If this law of handwriting had been respected, the fraction time for each symbol would have been approximately constant, independently from changes in size. This concept is highlighted by the shaded areas that comprise the range of fraction time variability between the spontaneous, big, and small executions. Wide areas mean that the corresponding symbol required a different fraction of the total sequence time, depending on the modality it was drawn. This is a violation of the invariance predicted by the homothety principle. The same happened for the rest of the population: subjects who scored greater than zero in the Homothety O-score had higher variability in fraction time, in respect to those who had an O-score equal to zero. In fact, the fraction time range for the circle, median [quartiles], was 0.08 [0.05; 0.13] for the zero-scoring group, and 0.1096 [0.0469; 0.2061] for the high-scoring group (Mann–Whitney *p*-value = 0.053); for the line it was 0.04 [0.03; 0.06] for the zero-scoring group and 0.05 [0.03; 0.13] for the high-scoring group (*p*-value = 0.011); for the reversed U it was 0.06 [0.04; 0.10] for the zero-scoring group and 0.13 [0.09; 0.25] for the high-scoring group (*p*-value < 0.001).

Panel d analyzes the speed-accuracy trade off (SAT), as formulated in the Steering law ^22^. The Steering law predicts a linear relationship between index of difficulty and movement time. The subject who achieved an O-score greater than zero in the SAT category (red) did not comply with the law, as shown by the non-significant regression line, with opposite 95% confidence intervals. Non-significant regressions were present only in children who obtained an O-score greater than zero in the Speed-Accuracy trade off feature category (TGS: 29.0%; TGW: 28.0%), whilst those who scored zero always achieved significance (Chi-squared *p*-value < 0.001).

**Figure 3:**
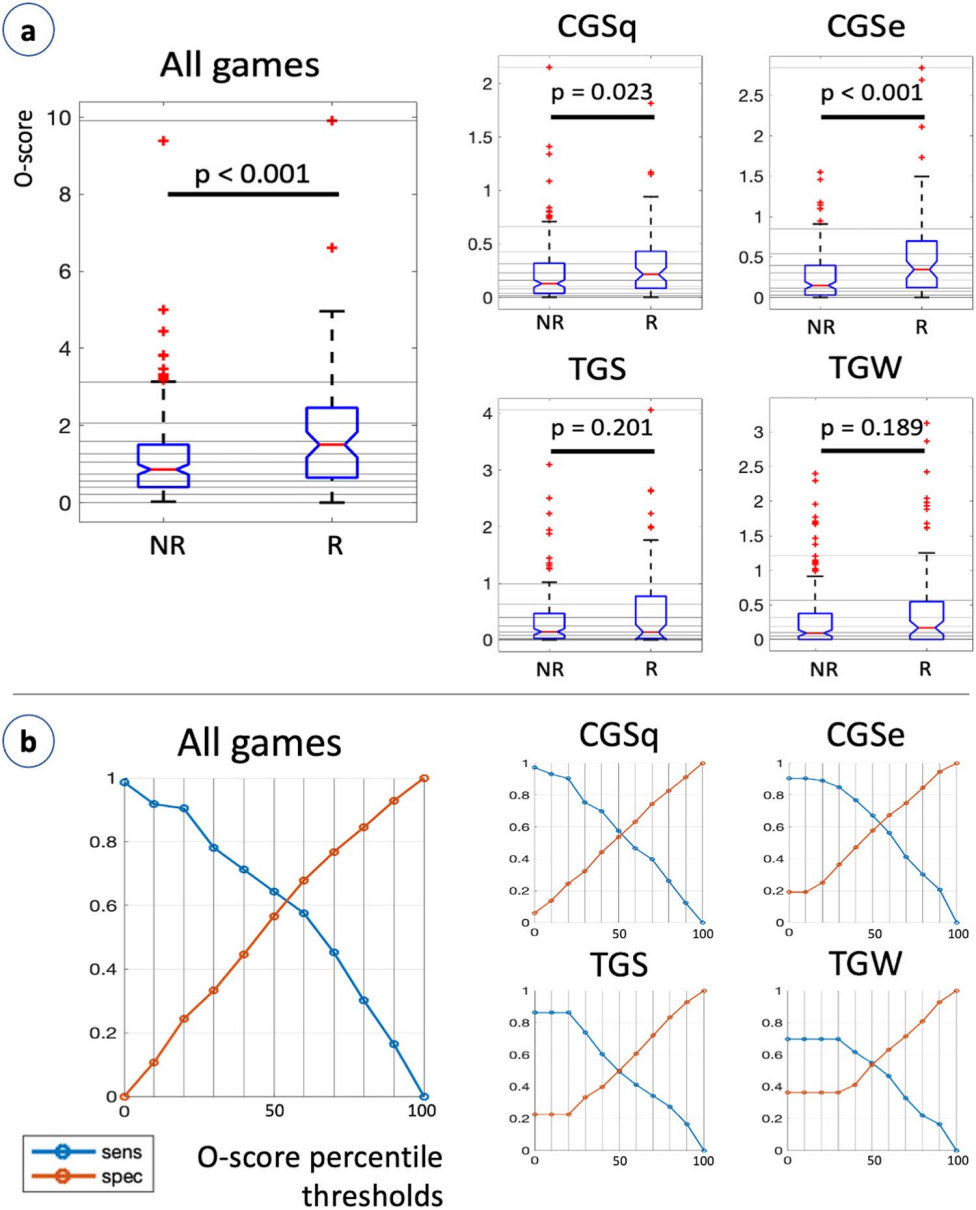
(**a**) O-score, stratified by risk. Red horizontal line: median; box: inter quartile range; notch: median 95% confidence interval; red crosses: outliers. The same graph is repeated considering all games together and each game separately. (**b**) Sensitivity and specificity at the different percentile thresholds. The same graph is repeated considering all games together and each game separately.

Figure 3a reports the distribution of the O-score, stratified by risk class. The Mann–Whitney U test revealed a significant difference in the O-score between R and NR children (*p*<0.001; NR median and quartiles: 0.86 [0.40, 1.50]; R: 1.503 [0.65, 2.46]). This means that R children tend to lay more often at the extremes of feature distribution (are more often outliers). This procedure was also applied to each game separately, to investigate whether a single game could result informative enough; in such a case, the protocol could be simplified to this specific task to become faster and increase adherence. We obtained again a significantly higher O-score for R children, compared with NR children, in the CGSq ($p=0.023$; NR: 0.13 [0.04, 0.32]; R: 0.22 [0.09, 0.43]) and in the CGSe (*p*<0.001; NR: 0.15 [0.03, 0.40]; R: 0.35 [0.12, 0.70]). This means that, in the R group, there was a higher incidence of performance that deviated from the standard. Significance was not reached in the TGS ($p=0.201$) and in the TGW ($p=0.189$).

The thresholds reported in Fig. 3a as horizontal lines are put at each tenth percentile of the O-score distribution. For each of these thresholds, sensitivity and specificity of the R children classification are reported in Fig. 3b Sensitivity declines to zero, as the threshold for being considered at risk becomes stricter (i.e., higher percentile). This procedure has been repeated for all games (i.e., all features) together, and considering only the features computed from each game separately.

**Figure 4:**
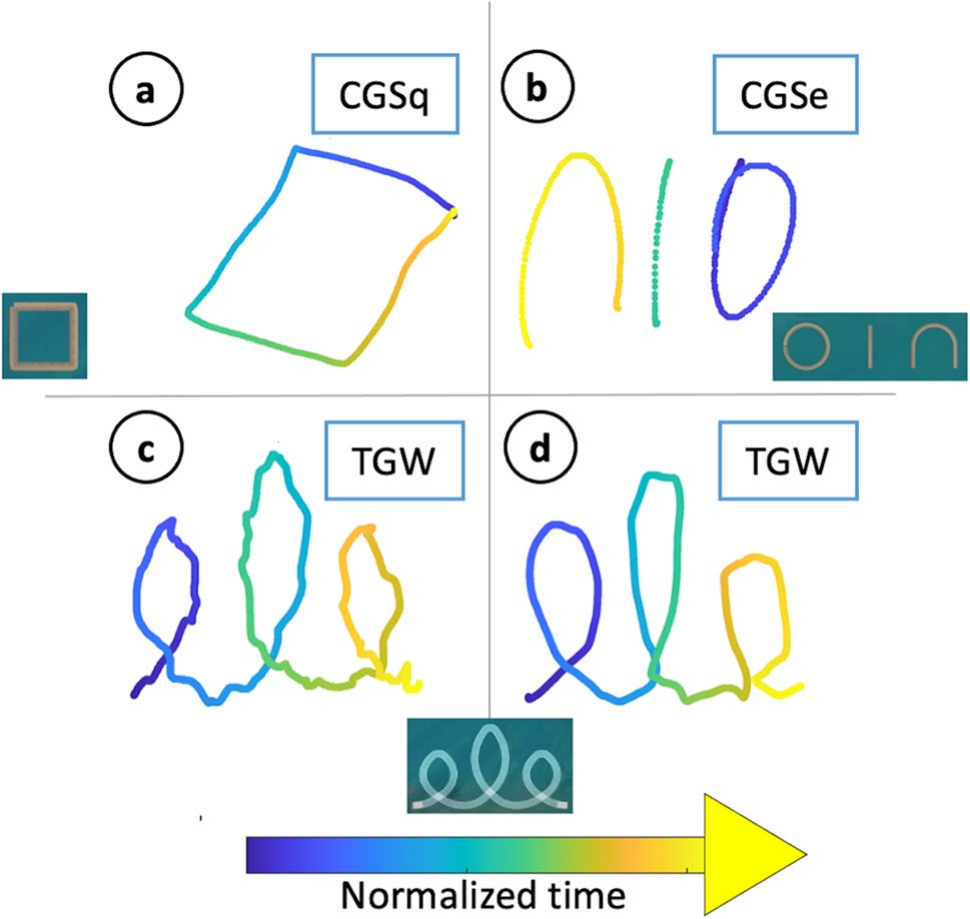
Examples of executions from NR children that achieved a high O-score. Color represents time progression. Templates are reported for reference.

From the result in Fig. 3a, the algorithm suggests that also some NR children tend to lay at the extremes of the feature distribution (high O-score). Hence, we took a deeper look at these children’s execution, to understand this phenomenon. Figure 4 shows the execution of four out of twelve children that teachers labelled as NR, but scored high (i.e., bad) with our algorithm (above the 90^th^ percentile of the O-score). Their executions present different kinds of abnormalities, that can motivate a high O-score, similar to R children. Panel a refers to CGSq, that was visibly rotated. Panel b refers to CGSe, but the sequence is both mirrored in respect to the template and drawn from right to left (color progression: from blue to yellow). Panels c and d refer to TGW. The first is really shaky, the second presents errors in the L and the second E loops (clockwise, without intersection).

We also analyzed the characteristics of the six R children who scored below the 10th percentile of the global O-score. All of them had difficulties in only one or two items of the questionnaire. One child had difficulties in locating differences between images (item 6); another one in recognizing rotated forms (item 7); a third one in both activities. One child had difficulties in occupying the space on the sheet (item 9); another one both in this activity and in steadily keeping the sheet while drawing (item 10). The last one had difficulties in respecting the correct drawing direction (item 11).

Considering the need for simplification—useful both for tests administration and for dimensionality reduction of the problem—, we proceeded in the analysis with the CGSe only, as it proved to be the most effective in the preliminary analyses.

### Risk detection

**Figure 5:**
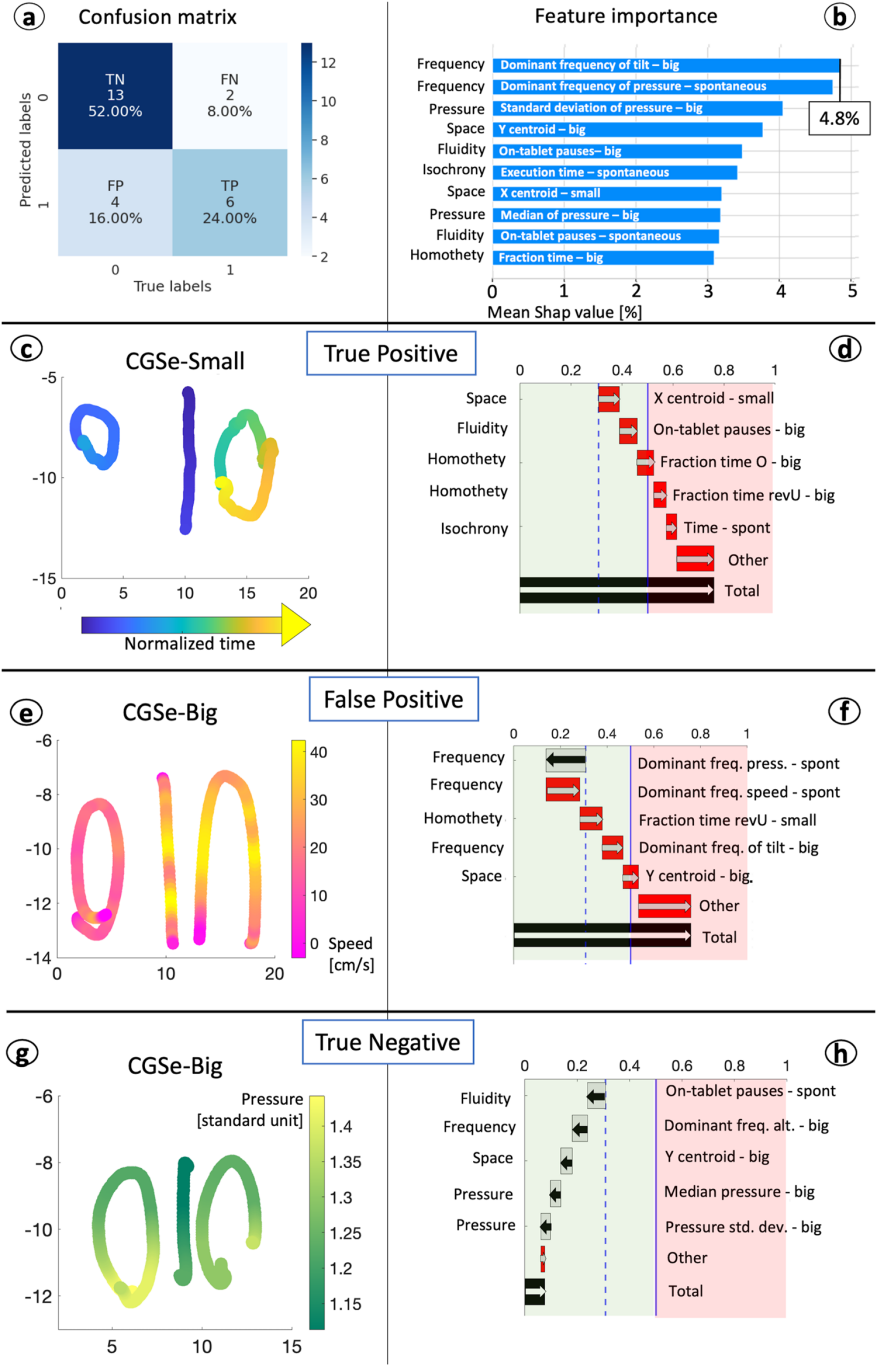
Results of the prediction. (**a**) confusion matrix of test predictions. (**b**) global normalized feature importance for the LearNet predictions on the test set, computed as Shapley values averaged on all subjects. (**c, e, g**) Sample executions. (**d, f, h**) The corresponding feature contribution to the prediction.

To classify a binary risk label, we leveraged machine learning techniques on CGSe features. Specifically, we designed a convolutional neural network, the LearNet, as described in the "Methods" section. As for features, maximum speed and maximum pressure were discarded, as they were co-linear with the corresponding median values. Hence, a total of 88 features were leveraged to train the model. Concerning participants, data from four children were discarded as they did not complete the game. As a result, the training set comprised 212 children, whilst the test set comprised 25 children. The structure of the LearNet, which resulted from Bayesian optimization, is shown in Table 1. The threshold computed to dichotomy the predicted probability was 0.5. The LearNet achieved a sensitivity of 75.0% and a specificity of 76.5% on the unseen test set. The confusion matrix is reported in Fig. 5a, whilst the comparison of the LearNet results with other classical machine learning models is reported in Table 2.

**Table 1:** Architecture of LearNet. The structure is intended to be sequentially ordered from top to bottom and divided into two parts: the first is composed of 1D convolutional layers and is intended as a feature extractor. The second is composed of dense layers and has the role of the classifier. We introduced a dropout between the first and the second dense layer to prevent overfitting.

Layer $\downarrow$	Units	Filters	Kernel size	Strides	Activation	Dropout probability
Conv1D	–	47	7	1	ReLu	–
Conv1D	–	57	2	2	ReLu	–
Conv1D	–	31	2	2	ReLu	–
Conv1D	–	21	7	2	ReLu	–
Conv1D	–	45	13	2	ReLu	–
Conv1D	–	39	9	2	ReLu	–
Flatten	–	–	–	–	–	–
Dense	53	–	–	–	ReLu	0.3
Dense	10	–	–	–	Sigmoid	–
Dense	1	–	–	–	Sigmoid	–

**Table 2:** Classification results. For each metric, the best performances is reported in bold.

Model	Accuracy	Sensitivity	Specificity	F1	G-Mean
Baseline (all NR)	68.00	0.00	$\mathbf {100.0}$	0.00	0.00
Logistic regression	64.00	25.00	82.40	30.80	45.40
Support vector machines classifier	60.00	12.50	82.40	16.70	32.10
LearNet	$\mathbf {76.00}$	$\mathbf {75.00}$	76.50	$\mathbf {66.70}$	$\mathbf {75.70}$

In Fig. 5, Panels c and e show the examples of executions belonging to two subjects that the model assigned to the R class. Even if one subject was a true positive (Panel c), and one a false positive (Panel e), the execution of both of them reveals abnormalities that probably guided the classification. In both, we can detect anomalies in the execution that justify the classification: in Panel c, symbols are coloured according to time, from blue to yellow, revealing that the progression was not left-right, as expected; in addition, the symbol furthest to the right presents a wrong shape. In Panel e, symbols are coloured according to the speed, that presents several fluctuations For reference, we report also a true negative (Panel g). The drawing is coloured according to the pressure exerted, that presents smooth changes between symbols parts; in addition, the shape of the sequence of symbols is correct.

We applied a feature ranking technique based on Shapley values ^23^ on the LearNet to explain how our model reached its performance. The ten features that mostly contributed to the prediction are shown in Fig. 5b. For each feature, the category is reported on the Y axis. The X axis represents the absolute mean of the impact of each feature with respect to the model default prediction. This means that the most important feature, i.e., *Dominant frequency of tilt-big*, has an average impact on the baseline prediction of 4.8% (see Fig. 5b). Figure 5 Panels d, f, and h show the individual Shapley value computed for the sample subjects reported in Panels c, d, and g, respectively. In this representation, starting from a 0.31 baseline computed by the algorithm, positive values (red bars) moved the prediction towards the R class, whilst negative values (grey bars) moved the prediction towards the NR class. The cumulative sum (black bar) is above 0.5 for subjects in panels d and e, as they were both classified as R, and below 0.5 for the subject in panel h, as she was classified as NR.

## Discussion

As failing to master handwriting have life-lasting consequences, an early intervention is fundamental. Hence, in this work, we provided solutions to anticipate the screening of handwriting disorders to a pre-literacy stage. We leveraged a tablet-based app, *Play-Draw-Write* ^21^, which allows investigating handwriting alterations typical of dysgraphia, but starting from symbol drawing. To assess the sensitivity of this tool, we designed an experiment that included 241 children. Children played the four games that compose *Play-Draw-Write*, that require copying or tracing symbols at different sizes or difficulties. Additionally, trained teachers performed a systematic observation of potential indicators of a graphical delay and filled in a 15-items checklist, that we considered the reference for the evaluation of our methods.

The sample was composed by approximately 30% of children considered at risk of developing an handwriting delay. This is in line with literature ^5^: high figures are common in early school years, and tend to decrease when proper training is performed ^7^. We can observe that the majority of children considered at risk presented difficulties in only few items, with a quasi-exponential decay when considering more items together. However, since correlation between items reaches significance only in few cases, we can infer that children presented heterogeneous difficulties, without many common patterns. This point stresses the necessity to address handwriting difficulties as a multi-dimensional problem ^10^.

Our first aim was to evaluate if playing with the app allowed disclosing pre-graphical difficulties. To this extent, we extracted objective features from game execution, and we transformed them into a score that summarized how often a child laid at the extremes of feature distribution (outlier score, O-score). We found that children considered at risk of a delay are more often outliers, with a significantly higher O-score. This expands the current scientific research on handwriting difficulties, that focused on describing anomalous behaviours from written words only ^9–11,16–20,24^. In the past, attempts of analysing the drawing of a circle provided poor results ^20^. This can be due to the nature of the features extracted (average speed, disfluency and average pressure), as the concept that the mere average of a kinematic or dynamic feature has been surpassed by recognizing that dysgraphic handwriting is characterized by high variability and unsteadiness ^9^. On the other hand, even when the analysis of lines and semicircles drawings seemed promising ^25,26^, the age of the target population (6-7 years old) did not guarantee the absence of an influence of handwriting knowledge in the drawings, thus impeding to translate the results to a pre-scholar age.

When stratifying by game the O-score, we observed that only the two Copy Games reached significance. Specifically, copying a sequence of symbols was the game where the distinction between children’s group was more evident. During such task, children were required to discriminate between symbols to correctly reproduce them in the given order, and to vary their size. Such task may result in a quite simple activity for children with age-adequate mastery of drawing. However, it involves good visual discrimination, working memory, ability to comply with space constraints, on top of simple graphical abilities. Given the fact that the analysis of the checklist responses shows that two of the most problematic items were Item 4 (copying a geometrical figure), and Item 11 (respecting the left-right directionality), this game seems to be the most complete to rank drawing proficiency. This can explain the higher predictive power shown by this exercise, in respect to the others, in an unsupervised risk detection. On the counterpart, the O-score computed on tunnel game features did not significantly differ between groups. We can conjecture that these games are demanding for all children, resulting in a flattening of the performance. To verify this point, older children should be assessed on these games, as it is recognized that speed-accuracy trade off abilities develop with age ^21^.

Reducing the problem to a single game allows simplifying the administration procedure. Hence, we proceeded considering only the Copy Game-Sequence to pursue our second goal, i.e., to detect children who mostly need to be helped. We trained a deep learning algorithm, the LearNet, to classify teachers’ labels, and we achieved 75.0% sensitivity and 76.5% specificity. It must be underlined that our model was trained on real-world data, that imply imbalanced class proportion, thus making the classification task even more challenging. This result further highlights the discrimination capability of the game, as similar classification tasks were recently applied on handwriting and produced comparable results ^11^. An additional achievement of our game design—that relies on symbols drawing—is the language-agnostic assessment. This represents a significant improvement from the current state-of-the-art, that is usually focused on specific languages or alphabets (e.g., Hebrew ^17^, French ^10^, or Slovak ^11^).

To achieve the third aim of suggesting a targeted intervention, we extracted the feature (and feature categories) in which children had difficulties, both from the O-score analysis and from the LearNet classification. When we stratified the O-score by category of features, we found a good correspondence with the raw execution, that is encouraging in terms of explainability.

This is important to shape a targeted intervention. As an example, if a child shows problems in the Pressure feature category, pressure modulation could be trained by proposing to draw with tools that allow to understand its change (pencil, wax crayon, pastel) and helping to visualize it with simple and playful examples (be heavy as an elephant, be light as a bird). The hand could be trained with manipulation games, such as, playing with clay, balling up paper, etc. Additionally, if a child shows problems in the drawing direction (Error feature category), it would be useful to train proprioception first, to understand the concepts of left and right on oneself body (e.g., swing an arm while the other one is still ^27^), and then suggest tracing paths or name figures following the correct direction, with the help of stories.

Moreover, our tool seems to be sensitive to deviations even when teachers did not report them: we examined the drawings from children considered not at risk, but with a high O-score, and we found out potential problems that justify the bad score. Therefore, our analysis highlights even aspects that cannot be noticed by a subjective evaluation only. Hence, our tool can be complementary to experts’ observation. This is promising, as *Play-Draw-Write* could become a valid aid in detecting areas of difficulties to be empowered, even without the need of a trained teacher, or even in a remote setting.

As for the deep learning model, from the average value of feature importance (Fig. 5b), it is evident that there is not a single feature the model relies on. Conversely, it learnt to exploit the information from different features jointly, and this is confirmed from both the absence of significant importance drops, and the low importance magnitude of the ten top features, which are able to change the baseline prediction only of few percentage points. Another explanation is that the important features are different for each subject, as confirmed by considering subject-wise feature ranking. This is in line with the heterogeneous picture of handwriting pre-requisites that we are considering for the Risk label. We can observe that the top-ranking features for the deep learning model belong to the Frequency category. This suggests that an alteration in the frequency domain is consistently distinctive of children with potential handwriting problems. The same holds when handwriting is analysed, as found in previous work ^9^. This confirms that the drawing process is similar to handwriting, thus enforcing the feasibility of an early screening.

The main limitation—which is also the main challenge—of this work is the uncertainty of the risk label. Even if teachers have been trained to assess children on a checklist co-designed by experts, by now it is not possible to assure if some of these children will improve or worsen in the future. Additionally, even if teachers underwent the same training, the evaluation may still be partially subjective, and depending on their usual class environment.

As an example, it could be argued that the risk imbalance found between the Italian and non-Italian mother-tongue children may be due to teachers’ pessimistic attitude towards the latter group. However, objective data collected with the *Play-Draw-Write* app confirmed that the latter group could be really disadvantaged, as supported by similar studies on kindergartners^28^, rather than being scored worse by teachers: a Mann–Whitney U test on the O-score (dependent variable) leveraging the Italian or non-Italian mother-tongue label as independent variable showed that the latter group performed worse (*p*-value = 0.016, median [quartiles]: 1.37 [0.67; 2.00]) than the first one (0.87 [0.42; 1.62]). However, confounding factors could have introduced additional noise into the labels. This is reflected in the results: we retrieved children with potential problems, even when some teachers discarded them.

The sharp discrimination between the two risk classes, that is inspired by classical diagnostic tests but may be affected by labels noise, can be instead modulated in a spectrum of severity, if considering the O-score or the SHAP score.

We are conscious that *Play-Draw-Write* cannot substitute the experts’ opinion. Instead, it should be an aid for an early intervention, suggesting a visit with a specialist for those children that do not improve in time. To address these points, a follow-up is scheduled in two-year time, when handwriting will be consolidated and a retrospective assessment will be possible. Moreover, within the European ESSENCE project ^29^, we are planning to leave the system at the children homes for one year to evaluate the feasibility of a periodic self-assessment and a fully automated risk evaluation.

To conclude, this work presents the potentiality of the *Play-Draw-Write* app in objectively detecting the risk of handwriting delay, starting from an age when handwriting is not learned yet. With our tool, we can (1) provide a ranking to prioritize the intervention, leaving to schools the decision on how many children to include in training programs, according to their capabilities; (2) provide a classification of children that could develop a handwriting delay, according to experts’ evaluation. In both cases, we are able to inform teachers—and professionals—of the specific weaknesses that should be trained. This is an important step towards the early treatment of handwriting difficulties.

## Methods

### Participants and protocol

241 children attending the last year of Kindergarten were included in the study. The sample size was computed considering the lower bound of dysgraphia incidence suggested in the literature (5%), with the aim of finding at least ten subjects at risk, with a 20% dropout. Subjects who could not understand Italian at all (just moved from a differently speaking country), or with a diagnosis of a neurological, visual, or motor pathology that can compromise drawing were excluded. Children from non-Italian mother-tongue families were included in the study, as long as they could understand the instructions. The information of belonging to families that moved to Italy from other countries—independently from the reason for moving—was retained, as it could be that parents who are unfamiliar with activities usually performed in kindergartens are less supportive in case of difficulties.

Children were asked to use the *Play-Draw-Write* app, with their preferred hand. First, they had to play the *Copy Game* (CG), that comprised coping a square (CGSq) and a sequence (CGSe) of a circle, a vertical line, and a reversed U. Three sizes were required: spontaneous, big, and small. Children were verbally instructed by the experimenter, who assured that the task was understood even by non-Italian mother-tongue children. However, symbols were not named, nor a tutorial was shown, as in similar neuropsychological tests ^30^. If children did not follow the requests (e.g., if they drew a flower or a cow instead of the square), they were asked to repeat the trial. Second, they had to play the *Tunnel Game* (TG) that comprised tunnels shaped as squares (TGS) and as the cursive sequence of letters “ele” (TGW), similarly to a dysgraphia test ^14^. For TG, verbal instructions were followed by a tutorial and an example trial. Then, children were requested to steer fourteen tunnels of different sizes, that corresponded to five indices of difficulty as in our previous work ^21^. During the trial of the “ele” tunnel, particular attention was payed to let them understand the correct direction (counterclockwise). Children had to repeat the trial if the pen was lifted before the end of the tunnel. An example of the four games is reported in the Supplementary Figure S1.

Data acquisition took place in a quiet room of the Kindergarten. Five Kindergartens, all located in the province of Varese (Italy), participated in the study. Acquisitions took place during January and February 2020. Data were collected by 6th and 7th generation iPads, with an Apple Pencil, 1st generation. The protocol was approved by Politecnico di Milano Ethical Committee, n. 24/2019, and an informed consent was signed by the children’s parents. The research was performed in accordance with relevant guidelines and regulations and in accordance with the Declaration of Helsinki.

### Risk evaluation by teachers

The definition of the *risk* of dysgraphia before handwriting is even learnt is nontrivial. Due to this complexity, previous studies leveraging drawings in handwriting skills assessment only recruited children who already mastered handwriting and could be more easily defined *at risk* ^25,26^, potentially altering the generalizability of the result to younger ages. In our work, to overcome this limit, starting from a structured review of the literature, a checklist was designed by a steering group of psychologists, child neuropsychiatrists, and bioengineers, with the aim of detecting the risk of handwriting delay.

Dysgraphia is a complex disability that does not simply affect handwriting quality ^10^. First, it is recognized that handwriting difficulties are associated with fine gesture impairment, such as cutting with scissors, touching fingers with the thumb, or screwing ^31^. In addition, visual discrimination capabilities could be a predictor of handwriting proficiency. Some examples can be the capability of matching rotated shapes, of copying a figure, of catching differences between images, or of understanding the concept of directionality ^32^. Then, inconsistent spacial occupation can often be found in this population, together with difficulties in achieving stability while writing. The latter may appear both as pressure fluctuations, and as coordination difficulties of the non-writing hand in holding the sheet steadily ^6^. Finally, the sense of rhythm—such as tapping, isochrony and homothety ^20^—and the capabilities of tracing in narrow paths are known to be altered in children affected by dysgraphia ^19^.

From this analysis, a 15-items checklist was developed, as reported in Supplementary Methods S2. The checklist was addressed to teachers who followed 10 days of a specific training program delivered by psychologists, child neuropsychiatrists, and pedagogists. They were required to observe children from the end of November 2019 to the end of January 2020, and to indicate if they had some difficulties in any item of the checklist. Each child was observed by two or three teachers, according to the kindergarten class structure, but a single judgment was agreed among them. Teachers were not influenced by *Play-Draw-Write* results, as they were blind to game execution that was performed by experimenters only.

We analyzed responses distribution with frequency analysis, and we computed the Spearman’s correlation between item responses to understand if some difficulty was often associated to others. Based on the checklist, a child was deemed at risk if he/she was reported to have difficulties in at least one item. We thus assigned a binary label of risk (R) and no-risk (NR) of a handwriting delay.

### Data processing

The *Play-Draw-Write* app sampled pen position, pressure, and tilt, with the associated timestamp, at 240 Hz. We leveraged Matlab R2020a to compute features that proved to be promising in discriminating between proficient and dysgraphic handwriting, as well as game-specific features.

Details on feature computation are reported in Supplementary Methods S3.

We distinguished them into ten categories, that are reported in Table 3. For each feature, Table 3 reports also the number of times it was computed. For the *Copy Game*, features were computed three times, once per level, with the exception of features that looked for trends over different levels, which were computed once. For the *Tunnel Game*, each feature was computed fourteen times, once per level, and then mediated when two or more levels shared the same index of difficulty ^21^, resulting in five repetitions of the same feature. The Speed-Accuracy trade off (SAT) features, that are computed over all levels combined, resulted in a single value. In total, 73 features were computed for the CGSq, 94 for the CGSe, 95 for the TGS and 97 for the TGW.

**Table 3:** Features extracted by the four games, with the number of repetitions.

Category	Feature	CGSq	CGSe	TGS	TGW
*Kinematics*	Median speed	x3	x3	x5	x5
	95th percentile of the speed	x3	x3	x5	x5
	Standard deviation of the speed	x3	x3	x5	x5
	Median acceleration	x3	x3	x5	x5
*Fluidity*	Signal-to-noise velocity peak diff.	x3	x3	x5	x5
	In-air time	x3	x3		
	On-tablet pauses	x3	x3	x5	x5
	Speed in crossing the ele intersection				x5
	Speed in the upper part of the ele loop				x5
	Speed diff. btw intersection and up loop				x5
*Angles*	Median tilt	x3	x3	x5	x5
	Standard deviation of the tilt	x3	x3	x5	x5
*Frequency*	Dominant frequency for the speed	x3	x3	x5	x5
	Dominant frequency for the pressure	x3	x3	x5	x5
	Dominant frequency for the tilt	x3	x3	x5	x5
*Pressure*	Median	x3	x3	x5	x5
	Maximum	x3	x3	x5	x5
	Standard deviation	x3	x3	x5	x5
*Space*	X centroid	x3	x3		
	Y centroid	x3	x3		
	Total trace length	x3	x3		
	Drawing strategy for the square${}^{\circ }$	x3			
	Std. dev. symbols height		x3		
*Errors*	Number of repetition of the level	x3	x3	x5	x5
	Wrong order in the sequence${}^{\hat{\,}}$		x3		
	Not drawing left-right		x3		
	Nr of out-of-tunnel points			x5	
	Out-of-tunnel time			x5	
	Out-of-tunnel speed			x5	
	Nr of ele loops performed clockwise				x1
	Nr of levels with clockwise loops				x1
*Isochrony*	Execution time	x3	x3		
	Time diff. divided by size diff.*	x2	x2		
	Size diff.*	x2	x2		
	Speed diff.*	x2	x2		
	Time diff.*	x2	x2		
	Speed diff. divided by size diff.*	x2	x2		
*Homothety*	Fraction time for each symbol		x3x3		
	Fraction time diff.*		x3x2		
*SAT*	Steering Law R$^{2}$			x1	x1
	Steering Law rmse			x1	x1
	Steering Law regression significance			x1	x1
	Steering Law Index of Performance			x1	x1
	Global movement time			x1	x1
	Level-wise movement time			x5	x5

${}^{\circ }$Starting point and the direction.

$^{\hat{\,}}$The final drawing did not replicate the given sequence.

${}^{*}$Difference between spontaneous and big levels, and between spontaneous and small levels.

### Graphical abilities analysis

To identify children deviating from their peers’ performance, we computed an “outlier score” (O-score) starting from the features in Table 3. For a robust estimation even with non parametric data, we used a median-based outlier detection method, with three scaled median absolute deviations as threshold ^33^. To compensate for the different number of outliers per feature, the binary score of being an outlier was weighted by the inverse of the number of outliers for each feature. Then, to achieve a final O-score that could approximate graphical abilities, single feature scores were summed up.

To check if the O-score can objectively model a graphical difficulty, for some feature categories we show exemplary executions that achieved an O-score equal to zero—that should correspond to good graphical abilities—and greater than zero—that should correspond to some weaknesses. Beyond the examples, representative features were chosen to prove that an O-score greater than zero corresponds to an alteration in graphical gesture production. To this end, we performed either Mann–Whitney U or Chi squared tests, by leveraging the chosen feature as dependent variable, and having an O-score equal to or greater than zero as independent variable.

To check if our approach found correspondence in teachers’ evaluation, we applied a Mann–Whitney U test on the O-score (dependent variable), stratifying children by risk (independent variable). Significance threshold was set to 0.05.

Then, we performed a sensitivity analysis. We considered different thresholds at each tenth percentile of the O-score distribution, considering all children together (both R and NR). For each threshold, we considered R children those above, and NR those below, and we computed sensitivity and specificity in the classification of R children at each threshold.

### Risk detection

To classify the risk of handwriting delay, we leveraged the previous analysis to choose the game that seemed to be more promising in modelling graphical abilities. The features computed in the chosen game were used as predictors to build a machine learning model.

It was preferred to use the whole set of features, and not only the O-scores in the different feature categories, as predictors because analysing more complex patterns may be helpful in risk detection. Hence, machine learning models directly applied on those features would be the right choice to uncover those patterns. Moreover, as the same features are computed on different game levels, also between-level patterns may be informative.

To avoid co-linearity, we computed pairwise Spearman’s correlation between features. If the 0.95 threshold was trespassed, one of the two features was removed from the feature set. Children not completing part of the selected game were discarded. Other missing values were imputed with the median or the mode values, for continuous or categorical features, respectively.

The whole dataset was divided into a train/validation set and test set, maintaining the NR/R proportion coming from the teachers’ evaluation. After that, both training and test sets were normalized on the training dataset, to minimize the bias. The model chosen for the prediction was a 1-dimensional convolutional neural network (1D-CNN). As the model was aimed at detecting learning difficulties in children, we named it LearNet. Such an architecture allows to find between-level patterns, instead of considering each feature separately. In addition, we set up the parameter search space in such a way that the receptive field could always cover the entire feature set, thus allowing all features to be linked together, regardless of their position in the input vector of the network. We replaced pooling layers with 2-step strides to maintain the whole information from the vector space, instead of restricting it as in 2D-CNNs, where feature spaces are much larger. Layer parameters were tuned via Bayesian optimization and evaluated by 10-folds stratified cross-validation. This resulted in ten models with identical configuration but trained and evaluated on different folds. We chose to combine Bayesian optimization with stratified 10-fold cross-validation because we believe it is a good trade-off between evaluation robustness (i.e., between holdout and nested k-fold cross-validation) and parameter search time (i.e., fixed parameters and wide grid search). Given the classes imbalance, it was not possible to optimize the parameter search with respect to conventional metrics, such as, accuracy or specificity. Then, the optimization metric chosen was the geometric mean, i.e., the square root of sensitivity and specificity, which is particularly suitable for unbalanced classification problems^34^. An optimization metric that accounts for both sensitivity and specificity allows to minimize the number of false negatives without negatively impacting true positive and true negative rates.

We guaranteed a wide exploration margin in terms of epochs for each model by setting a validation early stopping based on the geometric mean and with 200 steps of patience. Finally, the ten models obtained via the stratified 10-folds cross-validation were applied to the test set and the results were stored in terms of probability. An average probability was computed and data were dichotomized according to the threshold moving technique to deal with the classes imbalance ^35^. To assess the goodness of LearNet, we compared it against two classical machine learning models, i.e., Logistic Regression and Support Vector Machines Classifier. Regarding Support Vector Machines Classifier, we tested different kernel choices by grid search, finding that the best performance was obtained using the linear one.

As for our third aim, to explain the predictions, we used SHapley Additive exPlanations (SHAP) ^36,37^, a model explanation library based on game theory. It computes the Shapley values of the features, according to their impact on the model’s predictions. We applied SHAP to build the feature ranking for each model of the stratified 10-fold cross-validation, with respect to their predictions on the test set. Finally, we averaged each set of Shapley absolute values to obtain the final feature ranking.

LearNet and SHAP were implemented in Python 3.9 with Tensorflow 2.6.0.

## Supplementary Information

Supplementary Information 1.

Supplementary Information 2.

Supplementary Information 3.

## Data Availability

The data that support the findings in this study are available from the corresponding author upon reasonable request.

## Code availability

The code is available from the corresponding author upon reasonable request.
