# Supplementary material for: Identification and characterization of learning weakness from drawing analysis at the pre-literacy stage
Source: Sci Rep. 2022 Dec 14;12:21624. doi: 10.1038/s41598-022-26038-9 (PMC9749627; doi:10.1038/s41598-022-26038-9)
Supplement: Supplementary file 1 — Supplementary Information 1. [file 41598_2022_26038_MOESM1_ESM.pdf]

**Identification and characterization of learning weakness from drawing analysis at the pre-literacy stage**

Linda Greta Dui, Eugenio Lomurno, Francesca Lunardini, Cristiano Termine, Alessandro Campi, Matteo Matteucci, Simona Ferrante

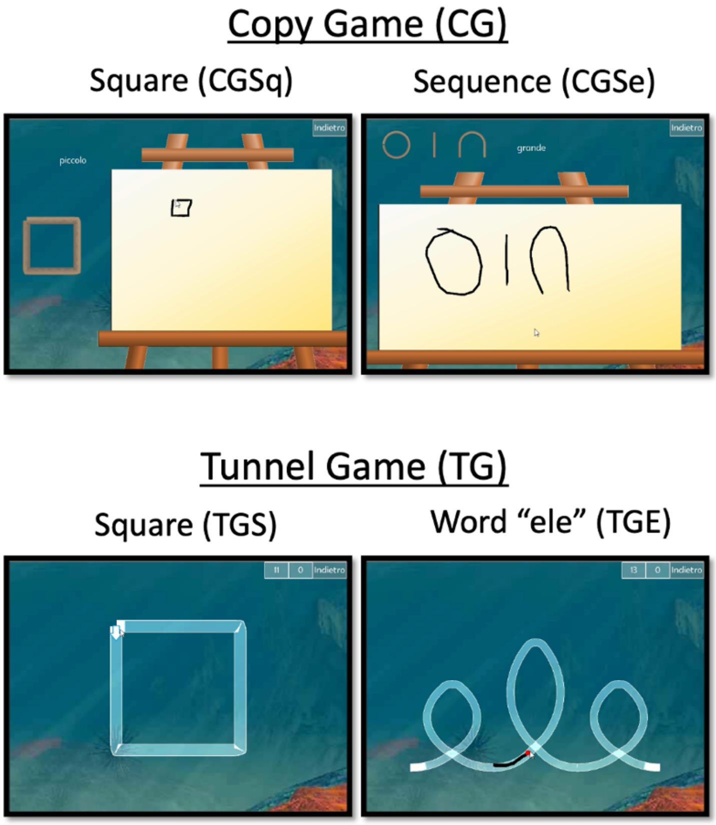


**Supplementary Figure S1**. Sample screens of the *Play-Draw-Write* games.
