# Supplementary material for: Identification and characterization of learning weakness from drawing analysis at the pre-literacy stage
Source: Sci Rep. 2022 Dec 14;12:21624. doi: 10.1038/s41598-022-26038-9 (PMC9749627; doi:10.1038/s41598-022-26038-9)
Supplement: Supplementary file 2 — Supplementary Information 2. [file 41598_2022_26038_MOESM2_ESM.pdf]

Identification and characterization of learning weakness from drawing analysis at the pre-literacy stage

Linda Greta Dui, Eugenio Lomurno, Francesca Lunardini, Cristiano Termine, Alessandro Campi, Matteo Matteucci, Simona Ferrante

Supplementary Methods S2. Checklist to evaluate the risk of developing handwriting delay.

Please, indicate if the child had a difficulty in one of the following activities:

1. Tracing a line in tunnels of 1 cm thickness;
2. Using the thumb to touch the other fingers (at least in the dominant hand);
3. Cutting along a straight line;
4. Copying a geometrical figure (square, triangle, rhombus, rectangle) in a recognizable way;
5. Adequately performing fine hand movements (screwing, unscrewing, ripping, balling up, wrapping a ball of yarn, knotting, unbuttoning the jacket, using the zipper...);
6. Locating differences and similarities between two images;
7. Recognizing rotated or inverted forms, in respect to a given model;
8. Reproducing rhythmical sequences by clapping the hands;
9. Occupying the whole sheet when drawing, upon request;
10. Steadily keeping the sheet with the non-writing hand, and correctly keeping the pencil while resting the writing hand wrist on the writing surface;
11. Respecting the right-left and up-down directionality in pre-graphical activities;
12. Exerting adequate pressure in pre-graphical activities (not too much, nor too few);
13. Autonomously writing oneself name;
14. Copying a simple word in block font;
15. Distinguishing letters from other graphical signs.
