# Supplementary material for: Identification and characterization of learning weakness from drawing analysis at the pre-literacy stage
Source: Sci Rep. 2022 Dec 14;12:21624. doi: 10.1038/s41598-022-26038-9 (PMC9749627; doi:10.1038/s41598-022-26038-9)
Supplement: Supplementary file 3 — Supplementary Information 3. [file 41598_2022_26038_MOESM3_ESM.pdf]

Identification and characterization of learning weakness from drawing analysis at the pre-literacy stage

Linda Greta Dui[1,*], Eugenio Lomurno[1], Francesca Lunardini[2], Cristiano Termine[3], Alessandro Campi[1], Matteo Matteucci[1], and Simona Ferrante[1]

[1]Department of Electronics, Information and Bioengineering, Politecnico di Milano, Milan, Italy
[2]Child Neuropsychiatry Unit, Fondazione IRCCS Istituto Neurologico Carlo Besta, Milan, Italy
[3]Child Neuropsychiatry Unit, Department of Medicine and Surgery, University of Insubria, Varese, Italy
[*]Corresponding author: Linda Greta Dui, MSc, Department of Electronics, Information and Bioengineering, Politecnico di Milano, Via Colombo 40, Milan, 20133, Italy. E-mail: lindagreta.dui@polimi.it

Supplementary Methods S3

In the following, the data processing to compute all the features is described in details sharing the feature in the following categories: kinematics, fluidity, pen angles, frequency, pressure, spatial occupation, errors, isochrony, homothety, Speed Accuracy Tradeoff (SAT).

Concerning the *kinematics category*, (x,y) positions in time were leveraged to compute the speed vector. The first step was to smoothen position data by applying a 20Hz lowpass filter[1]. Then, the raw speed was computed as in Equation 1

$$\sum_{n=1}^{N} \|\vec{p}(x(t),y(t)) - \vec{p}(x(t-1),y(t-1))\|/dt \tag{1}$$

where N is the number of sampled points and $\vec{p}$ is the position vector. To preserve vector length, the first computed value was replicated. Then, the speed was low-passed again to 10Hz. To further manage potential border effects introduced by filtering, the first and last 5% of the speed signal was discarded before computing as features the following summary values:

- median speed;
- the maximum speed, intended as the 95th of speed (the real maximum would have introduced noise)
- standard deviation of the speed;

Again in the kinematics category, the acceleration was computed by leveraging the discrete derivative of the speed. Hence, in this case, it is possible to have both positive and negative values. The feature computed from the acceleration vector was:

- median acceleration.

Concerning the *fluidity category*, the following features were computed:

- signal-to-noise velocity peak difference (SNVPD,[2]): the difference of the number of peaks in speed when applying a 10Hz low-pass filter, with respect to the number of peaks in speed when applying a 5Hz low-pass filter;
- in-air time[3]: the percentage of time spent with the pen lifted from the tablet, with respect to the total time of execution;
- on-tablet pauses: the percentage of time spent with the speed lower than the minimum between a speed of 0.05 cm/s or the lower 5% of the speed range;

Then, other three features fell into the fluidity category, but they are relevant only for the TG where the request was to steer the word:

- median speed when crossing the “ele” loops intersection;
- median speed when steering the upper part of the “ele” loops;

- speed difference between loop and intersection.

Concerning the *pen angles category*, following the literature[4,5], it was chosen to focus on tilt only computing:

- median altitude;
- standard deviation of the tilt.

Concerning the *frequency category*, the fast Fourier transform was computed and the frequency value where the spectrum had a peak was considered as the dominant frequency. This applies for:

- speed;
- pressure;
- altitude.

Concerning the *pressure category*, the pressure vector was leveraged to compute:

- median;
- maximum. This time, as the maximum pressure was bounded by the pen scale, it was not necessary to leverage percentiles.
- standard deviation;

Concerning the *spatial occupation category*, the features computed were:

- x and y centroids, computed as the median of the entire x and y vectors;
- trace length, computed as the sum of the norm of the vectors.

For the CGSq only, the x and y signals where leveraged to identify eight different patterns, that corresponded to different starting point and drawing direction. A ninth pattern was identified, as children were also allowed to copy the square by lifting the pen, side by side[6]. Indeed, a discrete feature was computed:

- drawing strategy.

For the CGSe only, to mimic spatial problems in dysgraphic handwriting[7], the features computed was:

- standard deviation of symbol height.

Concerning the *errors category*:

- the number of repetitions needed to complete the exercise. E.g., if the child lifted the pen before the end of the tunnel, the acquisition stopped; if the child drew an out-of-topic symbol instead of copying the square, she was asked to repeat it;

For the CGSe only, two kind of errors were identified:

- spatial errors: the final result did not match the pattern, i.e., the order of the symbols was incorrect;
- temporal errors: the left-to-right direction was not followed.

For the TGS only, three kind of errors were identified:

- percentage of the number of out-of-tunnel points;
- percentage of the time spent out of the tunnel;
- out of tunnel median speed.

For the TGW only, two kind of errors were identified:

- number of loops steered clockwise; it was the number of executions in which the “ELE” loop was carried out in the opposite direction, with respect to the correct way of writing the letters “ELE” in coursive;
- number of levels where at least one of the three loops was steered clockwise.

Concerning the *isochrony category*, it was relevant for the CGs only. The features included were:

- execution time;
- size difference, where the size was computed as the norm of the position vector $\vec{p}$ and the difference was computed between spontaneous and big modalities, and between spontaneous and small modalities;
- execution time difference divided by size difference (spontaneous-big, spontaneous-small);
- median speed difference (spontaneous-big, spontaneous-small);
- execution time difference (spontaneous-big, spontaneous-small);
- median speed difference divided by size difference (spontaneous-big, spontaneous-small);

Concerning the *homothety category*, it was relevant for the CGSe only. The features computed were:

- the fraction time for each symbol;
- the fraction time difference between modalities (spontaneous-big, spontaneous-small).

Concerning the *SAT category*, it was relevant for the TGs only. The features computed were:

- the R^2 of the linear regression between movement time (MT) and index of difficulty (ID), see Equation 2;

$$MT = a + b\,ID \tag{2}$$

- the root mean square error of the regression;
- the index of performance (IP) computed as the inverse of the regression line slope b, as in Equation 3;

$$IP = \frac{1}{b} \tag{3}$$

- the global movement time, mediated across all the repetitions;
- the level-wise movement time.

References

1. Van Galen, G. P., Van Doorn, R. R. & Schomaker, L. R. Effects of motor programming on the power spectral density function of finger and wrist movements. *J. Exp. Psychol. Hum. Percept. Perform.* **16**, 755 (1990).

2. Danna, J., Paz-Villagrán, V. & Velay, J.-L. Signal-to-noise velocity peaks difference: A new method for evaluating the handwriting movement fluency in children with dysgraphia. *Res. developmental disabilities* **34**, 4375–4384 (2013).

3. Rosenblum, S., Weiss, P. L. & Parush, S. Handwriting evaluation for developmental dysgraphia: Process versus product. *Read. writing* **17**, 433–458 (2004).

4. Asselborn, T. *et al.* Automated human-level diagnosis of dysgraphia using a consumer tablet. *NPJ digital medicine* **1**, 1–9 (2018).

5. Mekyska, J. *et al.* Identification and rating of developmental dysgraphia by handwriting analysis. *IEEE Transactions on Human-Machine Syst.* **47**, 235–248 (2016).

6. Frankenburg, W. K., Dodds, J., Archer, P., Shapiro, H. & Bresnick, B. The denver ii: a major revision and restandardization of the denver developmental screening test. *Pediatrics* **89**, 91–97 (1992).

7. Hamstra-Bletz, L., DeBie, J., Den Brinker, B. *et al.* Concise evaluation scale for children's handwriting. *Lisse: Swets* **1**, 623–662 (1987).
